# Challenges of acute febrile illness diagnosis in a national infectious diseases center in Rio de Janeiro: 16-year experience of syndromic surveillance

**DOI:** 10.1371/journal.pntd.0011232

**Published:** 2023-04-03

**Authors:** Clarisse da Silveira Bressan, Maria de Lourdes Benamor Teixeira, Maria Isabel Fragoso da Silveira Gouvêa, Anielle de Pina-Costa, Heloísa Ferreira Pinto Santos, Guilherme Amaral Calvet, Otilia Lupi, Andre Machado Siqueira, Rogério Valls-de-Souza, Clarissa Valim, Patrícia Brasil

**Affiliations:** 1 Acute Febrile Illnesses Service, Oswaldo Cruz Foundation, Rio de Janeiro, Rio de Janeiro, Brazil; 2 Laboratory of Epidemiology Research and Social Determinants of Health, Oswaldo Cruz Foundation, Rio de Janeiro, Rio de Janeiro, Brazil; 3 Universidade Federal Fluminense, Niterói, Rio de Janeiro, Brazil; 4 Department of Global Health, Boston University School of Public Health, Boston, Massachusetts, United States of America; National Institute of Health, INDIA

## Abstract

**Introduction:**

Acute febrile illnesses (AFI) are a frequent chief complaint in outpatients. Because the capacity to investigate the causative pathogen of AFIs is limited in low- and middle-income countries, patient management may be suboptimal. Understanding the distribution of causes of AFI can improve patient outcomes. This study aims to describe the most common etiologies diagnosed over a 16-years period in a national reference center for tropical diseases in a large urban center in Rio de Janeiro, Brazil.

**Methods:**

From August 2004-December 2019, 3591 patients > 12 years old, with AFI and/or rash were eligible. Complementary exams for etiological investigation were requested using syndromic classification as a decision guide. **Results**. Among the 3591 patients included, endemic arboviruses such as chikungunya (21%), dengue (15%) and zika (6%) were the most common laboratory-confirmed diagnosis, together with travel-related malaria (11%). Clinical presumptive diagnosis lacked sensitivity for emerging diseases such as zika (31%). Rickettsia disease and leptospirosis were rarely investigated and an infrequent finding when based purely on clinical features. Respiratory symptoms increased the odds for the diagnostic remaining inconclusive.

**Conclusions:**

Numerous patients did not have a conclusive etiologic diagnosis. Since syndromic classification used for standardization of etiological investigation and presumptive clinical diagnosis had moderate accuracy, it is necessary to incorporate new diagnostic technologies to improve diagnostic accuracy and surveillance capacity.

## Author summary

Different etiologies of acute fever may have an unspecific clinical presentation, especially in the early days of onset. Urban centers in low-and middle-income countries share the reality of overloaded healthcare systems—especially during outbreaks–whose diagnostic capacity is limited. Even in reference centers, determining the etiology of acute fever may not be possible in a large proportion of patients. The incidence of pathogens other than Malaria and Arboviruses is scarcely reported in Latin America. In cities where Dengue Fever is highly endemic, management of acutely ill patients is jeopardized by the unfamiliarity of health professionals with the spectrum possible diagnosis. Therefore, learning about the distribution of different etiologies of fever in outpatient services can impact positively in patient care. Since specific laboratory tests are determinant for differentiating among etiological agents of fever, further improvement for point-of-care diagnosis, as well as their availability for the front-row assistance settings is essential. This study aims to describe the most common etiologies of Acute Febrile Illnesses observed over a 16-year period time inside one of the largest reference centers for tropical diseases research in Brazil, discussing the accuracy of presumptive clinical diagnosis and the challenges of laboratorial investigation.

## Introduction

Fever is a common complaint in patients seeking health care worldwide [1] and etiological diagnosis of acute febrile illness (AFI) is difficult. Different etiologies may have an overlapping and unspecific clinical presentation, especially in the early days of onset. Furthermore, in large urban centers of low- and middle-income countries (LMICs), healthcare systems are overloaded—especially during epidemics and outbreaks–and diagnostic capacity for AFIs has always been limited by the uneven availability of laboratory tests [2].

Accurate diagnosis of AFIs is also difficult when there is endemic transmission of highly prevalent agents, which leads providers to automatically attribute every cause of fever to the predominant pathogen. For instance, in cities where dengue is highly endemic, primary care physicians frequently misdiagnose other AFIs as arboviral diseases. Management of AFI patients can be jeopardized by the unfamiliarity of health professionals with the spectrum of infectious diseases to which inhabitants and travelers may have been exposed, as it occurs outside the endemic areas. Learning about the distribution of different etiologies of acute fever in outpatient services can improve patient care.

Rio de Janeiro (RJ) is a touristic and trade hub that hosts several international events. Therefore, the city is vulnerable to the emergence of imported epidemics. Also, it is a major urban center inside a resource-limited country and highly endemic for several tropical diseases, such as tuberculosis, congenital syphilis, leptospirosis, chikungunya and all four serotypes of dengue. Laboratory diagnosis is often delayed or performed retrospectively, and the true incidence of these infections is many times underestimated. Study of the variety of etiological agents circulating in RJ can improve the ability to diagnose acute fever in this city and in other similar urban centers, optimizing early diagnosis and the detection of emerging new infectious diseases.

The aim of this study is to describe the clinical presentation and etiologic diagnosis of AFI in outpatients who sought care over a 16-year period in a reference center for tropical diseases in Rio de Janeiro.

## Methods

### Ethics statement

The study protocol was approved by the INI-Fiocruz Research Ethics Committee (CAAE 88551218.6.0000.5262). All participants or their legal guardians gave written informed consent prior to the study entry and data collection followed strictly international ethical standards of Good Clinical Practice.

### Study site

This study was performed at the Acute Febrile Illness Outpatient Clinic (AFIOC) in Evandro Chagas National Institute of Infectious Diseases, (INI-Fiocruz), one of the units of Hospital-based Epidemiological Surveillance of Brazil´s Ministry of Health (MoH). The AFIOC diagnoses are mostly supported by the National Laboratories of Reference from Fiocruz (Flavivirus, Hantaviruses and Rickettsiae, Respiratory Virus, Malaria), which allows the investigation of reportable diseases and emerging and reemerging pathogens. Together we do the surveillance of dengue and other arboviral diseases, leptospirosis, rickettsiosis, and malaria, and assist the detection of local transmission of new pathogens including Zika, H1N1 epidemic influenza virus, and SARS-CoV-2. Due to its central location, AFIOC provides care to neighboring communities and has become a reference center to basic health units, secondary and tertiary hospitals located inside and outside Rio de Janeiro municipality.

### Study population

All patients older than 12 years of age, who sought care from August 2004 to December 2019, were included in this report if they had a self-reported fever and/or skin rash in the previous seven days, or had indefinite time of fever if associated with travel to an area with malaria transmission.

Patients were excluded from this report if they had suspected tropical diseases causing fever lasting more than a week; presumptive noninfectious conditions; and insufficient clinical and laboratory information.

### Study procedures

#### Clinical evaluation and data collection

The medical assessment included sociodemographic and epidemiologic information in addition to routine medical history and physical examination, all recorded in a standardized case report form. At the end of the appointment, a clinical hypothesis was formulated based on clinical judgement of the attending physician. For the purpose of this study, we have described only clinical features shown at the first medical appointment.

#### Etiologic investigation

All patients provided blood samples for routine laboratory tests, including full blood cell count and biochemistry, along with specific tests: dengue NS1 antigen (Dengue NS1 AG Atrip, BIO RAD or Dengue Duo Test, BIOEASY) and dengue serology (Dengue IgM ELISA capture PANBIO, Dengue Indirect IgG ELISA, PAN BIO). After 2015, aliquots of blood and urine were also routinely used for dengue, zika and chikungunya virus detection, using real-time reverse transcriptase polymerase chain reaction assay (RT-PCR); and Chikungunya IgM and IgG antibody serology as described [3].

Further complementary exams for etiological investigation were requested using syndromic classification as a decision guide, a routine procedure in the AFIOC since 2004 (S1 Fig). These might include bacterial culture of body fluids; thick and thin blood smear for malaria diagnosis; serological tests for acute and convalescent antibody detection against cytomegalovirus, toxoplasmosis, Epstein-Barr, varicella, measles, parvovirus B19, rubella, microagglutination tests for leptospirosis (MAT) and indirect immunofluorescence (IFI) for rickettsia disease (Brazilian Spotted Fever). Until 2019, exams for detection of respiratory viruses by RT-PCR were not available for routine care, except during outbreaks such as the H1N1 epidemic in 2009.

All the above-mentioned molecular tests were performed according to routine protocols previously defined by the respective National Reference Laboratories of Fiocruz [4–6].

#### Diagnostic criteria

At the end of the study, participants were classified according to a definitive etiologic diagnosis based on expert consensus review of laboratory, clinical and epidemiological data (S1 Table). We considered as laboratory-confirmed dengue a patient who had either a positive NS1 antigen test or a detected dengue virus by RT-PCR, or viral isolation. For those patients who attended the clinic before December 2014, laboratory-confirmed dengue was also based on presence of at least one of the following: detection of IgM antibodies in serum after five days of the beginning of clinical symptoms; IgG seroconversion, or a four-fold raise in IgG antibodies titers in the convalescent phase.

Zika virus (ZIKV) infections were determined by positivity of RT-PCR in serum, urine or cerebrospinal fluid specimens. A definitive etiologic diagnosis of chikungunya virus infection was based on presence of one of the following: a positive RT-PCR in serum, urine or cerebrospinal fluid, detection of IgM in serum after five days of the beginning of clinical symptoms; IgG seroconversion, or a four-fold raise in IgG antibodies titers in convalescent phase.

Malaria cases were confirmed by point-of-care (POC) thick and/or thin positive blood smears. Malaria rapid diagnostic test (RDT) was not taken into consideration alone to confirm the diagnosis.

Highly suspected cases who did not meet the above criteria were classified as “without diagnosis”.

### Statistical analyses

The frequency of sociodemographic and clinical features, were compared across groups through chi-square tests. Associations between the presence of clinical and laboratorial characteristics and the “without diagnosis” category was based on odds ratios (ORs) and the corresponding 95% confidence interval estimated in a binary logistic regression. P-values ≤0.05 were considered statistically significant. All statistical analyses were performed using the R software (version 4.1.1).

## Results

**Fig 1:**
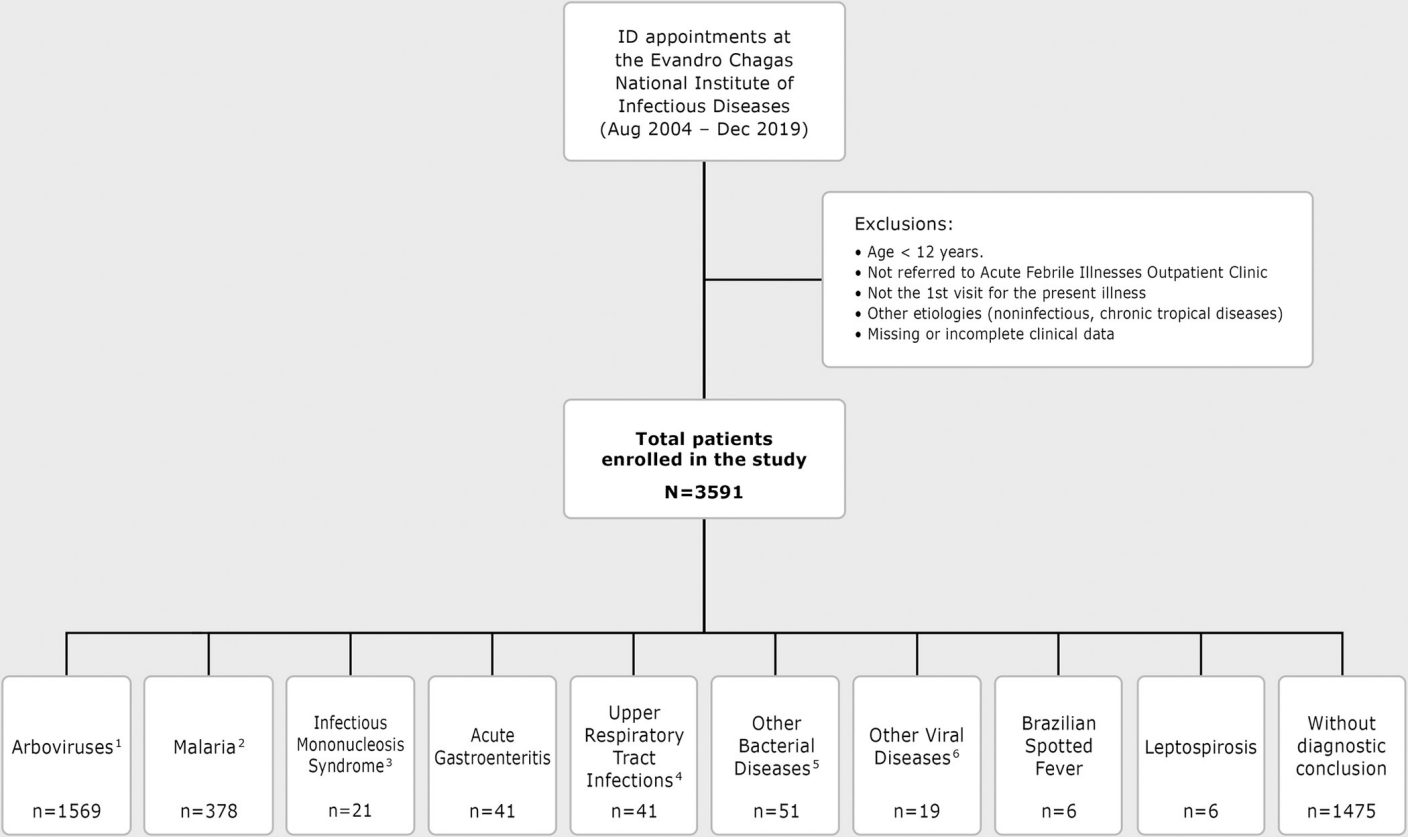
Flowchart with patient enrollment and the acute febrile illness etiologies from August 2004 to December 2019. 1. Laboratory-confirmed dengue (521 patients), zika (227), chikungunya (759), yellow fever (25); co-infections: dengue/zika (1), dengue/chikungunya (12), zika/chikungunya (6), dengue/malaria (7) and chikungunya/malaria (11). 2. Lab-confirmed *P*. *vivax* (248), *P*. *falciparum* (97), *P*. *malariae* (3), *P*. *ovale* (5), mixed (*P*. *vivax* and *P*. *falciparum*) (2); co-infection malaria/dengue (7), co-infection malaria/chikungunya (11), not specified (5). 3. Lab-confirmed acute infection caused by Epstein-Barr Virus (EBV), cytomegalovirus (CMV), HIV or *Toxoplasma gondii*, resulting in acute infectious mononucleosis syndrome. 4. Includes common cold and acute bacterial rhinosinusitis. 5. Clinical or lab-confirmed bacterial pneumonia (15), bacterial pharyngitis/tonsillitis (11), secondary syphilis (9), urinary tract infection (7), tuberculosis (3), typhoid fever and sepsis (2), cholecystitis (3) and brucellosis (1). 6. Acute viral hepatitis (A and B) (11), parvovirus B19 (3), Varicella-Zoster infection (including chickenpox) (4), nonspecific viral meningitis (1).

Among the individuals who sought care at AFIOC between August 2004 and December 2019, a total of 3591 patients were included in this study (Fig 1). The median age was 38 years (interquartile range [IQR] = 28, 50). Travelers corresponded to 39% of patients, with 9% coming from the Brazilian Amazon Region (Table 1). Among the 328 returning international travelers, 236 had visited Africa (72%). The interval between the onset of symptoms and seeking medical attention was less than seven days in 80% of the patients, while 12% arrived between eight to 14 days after the beginning of symptoms.

**Table 1:** Sociodemographic and epidemiologic characteristics of 3591 included patients from the Acute Febrile Illnesses Outpatient Clinic of the Evandro Chagas National Institute of Infectious Diseases, Rio de Janeiro, Brazil. (August 2004—December 2019) ^1^.

Characteristic	No.	(%)
**Age group (years)**		
12 to 18	180	5
19 to 40	1865	52
41 to 60	1235	34
≥61	311	9
**Gender (female)**	1760	49
**Race**		
White	2075	58
Non-White	1101	31
**Education (years of schooling)**		
<8 years	695	19
9–12 years	1131	32
>12 years	1526	43
**Travelled in the last 30 days**	1387	39
**Travel destination** ^2^		
Southeast Brazil (including RJ and Atlantic Forest Areas)	598	17
North of Brazil (including the Amazon Region)	332	9
Brazil (other States)	111	3
Central and South America	58	1.6
North America	8	0.2
African continent	236	7
Asian Continent	10	0.3
Europe	16	0.4
**Contacts with patients with similar symptoms**	1450	40
**Concomitant health conditions**		
Diabetes	184	5
Allergic rhinitis	545	15
Hypertension	598	17
HIV-infection	400	11
Previous dengue	1170	33
Smoking (current)	481	13
Alcohol (current)	948	26
**History of yellow fever vaccination**	1269	35
**Regular use of insect repellent**	186	5
**Time of symptom onset prior to the first visit (days)**		
≤7	2848	79
8 to 14	433	12
15 to 30	182	5
>31	76	2

^1^ Calculations of proportions excluded few subjects who were missing information in the specific question.

^2^ Travel destination information was available only for 1369 patients.

### Etiologic diagnosis

**Fig 2:**
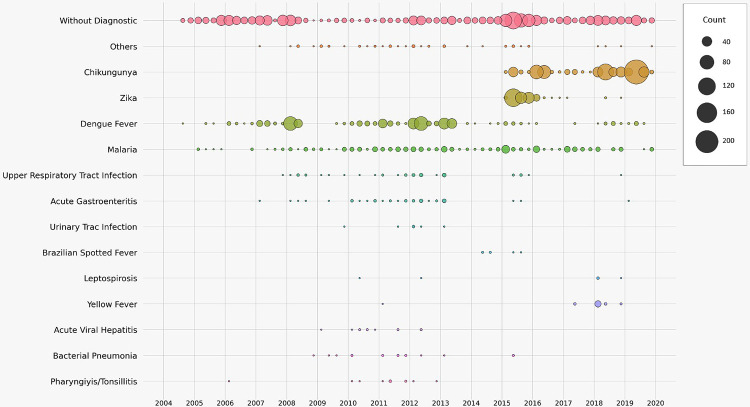
Etiologic diagnosis of the Acute Febrile Illnesses (AFI) over time, between August 2004 and December 2019 (N = 3591).

After complete laboratory investigation, dengue, zika and chikungunya were the most frequent diagnoses among patients treated at AFIOC and, together with a small number of patients with yellow fever enrolled during an outbreak, corresponded to 44% of the total outcomes. During the study timeline (Fig 2), the incidence of dengue has decreased as zika and chikungunya were introduced in 2015 and 2016. Co-infections of dengue and zika (n = 1), dengue and chikungunya (n = 12), zika and chikungunya (n = 6), chikungunya and malaria (n = 11) and dengue and malaria (n = 7) were found in a small number of patients. The diagnosis of Brazilian spotted fever, caused by *Rickettsia rickettsii*, was confirmed in 14% (n = 6) of the 42 suspected cases of this disease. The diagnosis of leptospirosis was suspected in 32 outpatients, but confirmed in six. Malaria was the final diagnosis in 11% (n = 378) of patients and *Plasmodium vivax* was the predominant etiologic agent (66%), followed by *P*. *falciparum* (26%), *P*. *ovale* (1%), and *P*. *malariae* (0.8%).

In 41% of the study participants, the laboratorial etiological investigation was inconclusive and patients remained without a definitive diagnosis.

### Signs and symptoms associated with an established etiologic diagnosis

**Fig 3:**
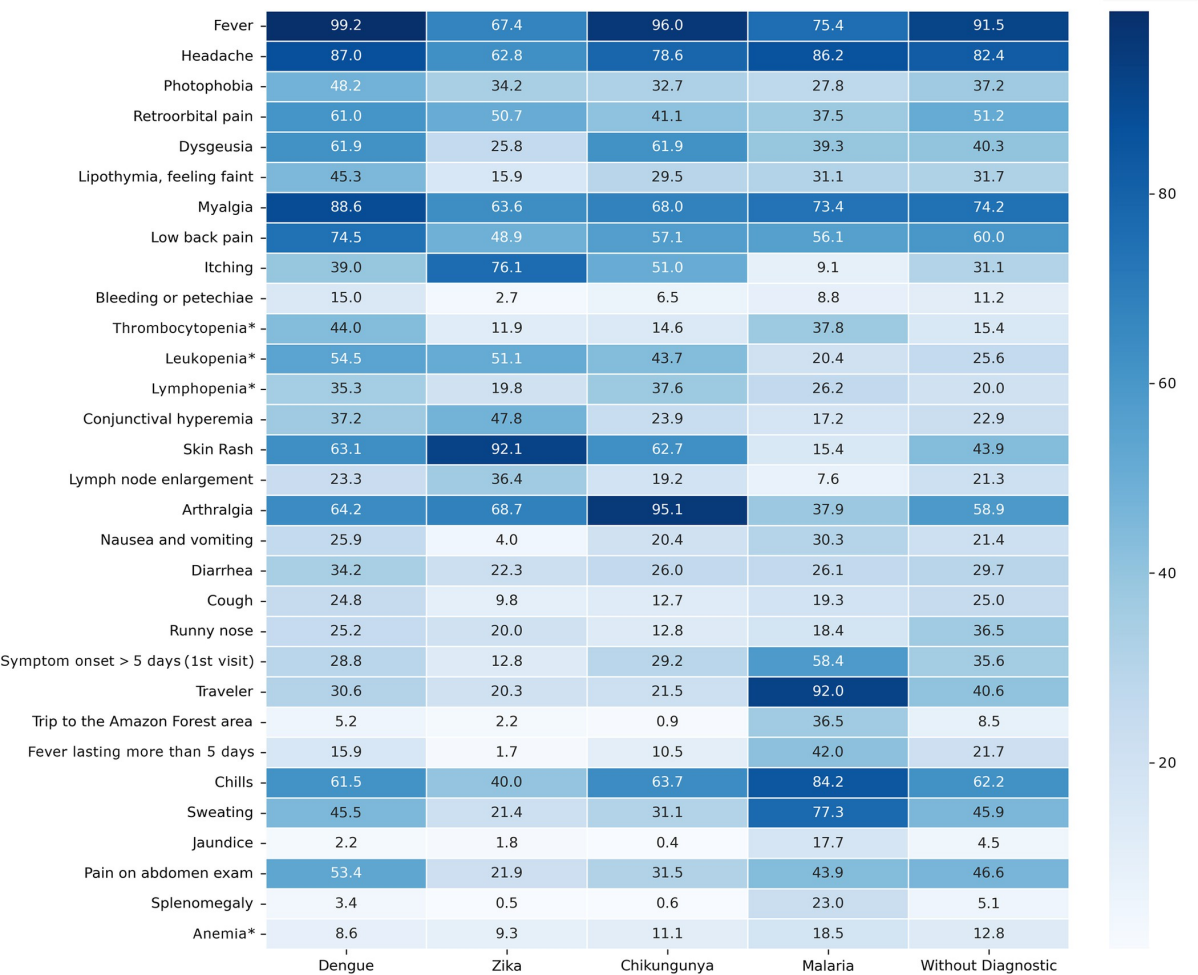
Comparison of the frequency (%) of clinical and laboratory findings reported at the first visit, according to the result of the etiological investigation (N = 3360). Co-infections and diagnosis with n ≤ 200 not included in this clinical description. Chi-square test showed significant differences among study groups: p<0.001 for all variables except for diarrhea (p = 0.003). * See S1 Table for the definition of anemia, leukopenia, lymphopenia and thrombocytopenia definitions used.

Fever was observed in all patients with dengue, and headache, myalgia and low back pain were the most common symptoms among confirmed cases (Fig 3). In contrast, 67% of patients with confirmed ZIKV infection reported fever, but commonly reported rash, itching, and arthralgia. In chikungunya, the most frequently reported symptoms were fever, arthralgia, headache and myalgia. In patients with malaria, headache, chills, sweating and previous travel report were the most frequent findings.

**Fig 4:**
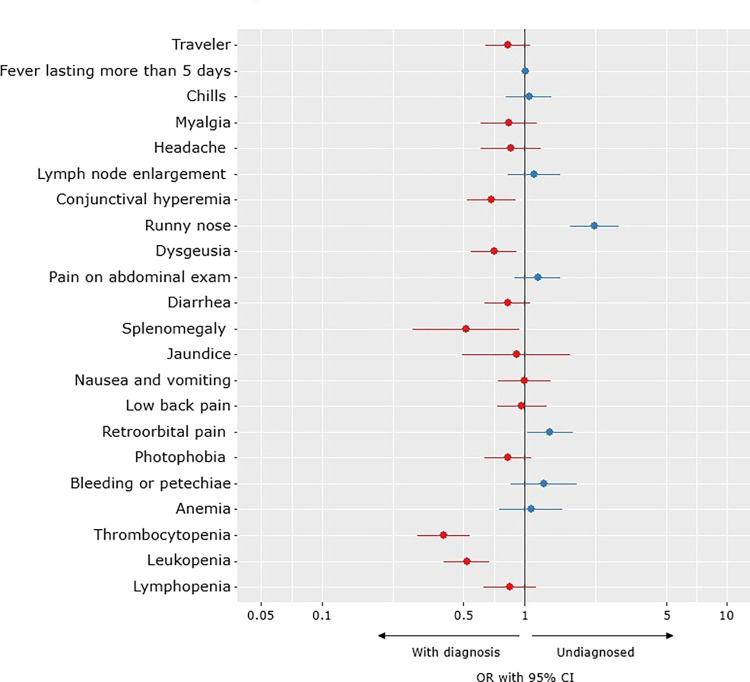
Clinical Features associated with the absence of a definitive diagnosis for the Acute Febrile Illness (AFI).

Among the patients without a definitive etiological diagnosis (“without diagnosis”), fever, headache, and myalgia were the most commonly reported symptoms. The presence of coryza and retroorbital pain were associated with absence of definitive etiologic diagnosis conclusion, while thrombocytopenia, leukopenia, dysgeusia, ocular congestion and splenomegaly were the most common findings in patients who had an etiological agent identified (Fig 4).

### Accuracy of the initial clinical diagnosis

At the end of first consultation, the attending physician diagnosed (“presumptive diagnosis”) dengue in 49% of the patients, zika in 7%, and chikungunya in 19% (Table 2). Among those, the sensitivity of the clinical presumptive diagnosis was 92% for dengue, 31% for zika and 71% for chikungunya; and the specificity was 58%, 95% and 95%, respectively. The positive predictive value (PPV) of the clinical presumptive diagnosis of dengue was 27%, and the negative predictive value (NPV) was 98%. For zika and chikungunya, PPVs were 35%, and 78%, respectively, and NPVs were 94% and 92%, respectively. For malaria, the sensitivity and specificity of the presumptive diagnosis was 89% and 95%, with a PPV of 67% and NPV of 99%.

**Table 2:** Most frequent presumptive diagnosis after the first medical appointment (N = 3401).

Presumptive diagnosis	No.	(%)
Dengue	1761	49
Chikungunya	685	19
Malaria	474	13
Zika	263	7
Infectious mononucleosis syndrome	45	1
Brazilian spotted fever	42	1
Upper respiratory tract infection	41	1
Leptospirosis	32	1
Rubella	26	1
Yellow fever	18	1
Viral hepatitis	14	0

## Discussion

Since 1981, when DENV was introduced in Brazil, it has become endemic in an increasing number of locations, ultimately leading to epidemic waves in urban centers in 1986, 1991, 1998, 2002, 2008, 2010, 2015 and 2019 [7]. Severe dengue cases have been increasingly reported since 2006, especially among children and young adults, as the country becomes hyperendemic [8]. In the present study, dengue was by far the predominant diagnosis for almost a decade among study participants, with ups and downs reflecting epidemic and endemic periods in the RJ municipality [9]. After 2015, the incidence of zika and chikungunya abruptly increased, following the introduction of these arboviruses in RJ, while the reported incidence of dengue cases decreased.

The PPV of the presumptive clinical diagnosis of dengue in this study was lower than in similar studies carried out in the same institution before the introduction of zika and chikungunya [10], reflecting the diagnostic challenge of these diseases with similar clinical presentation even for experts in infectious diseases. The zika PPV was similarly low, especially compared to that of chikungunya. These figures suggest that the diagnosis of dengue was more often mistaken with zika than with chikungunya.

The most frequent clinical features in the first consultation of patients with a confirmed etiology were those found in AFIs in general. However, we identified some symptoms and signs that may help to distinguish the three commonly diagnosed arboviruses. There was a greater number of patients without fever and with a pruritic rash among those with laboratory-confirmed ZIKV infection than in those with other arboviruses infections, in agreement with a previous study [11]. Arthralgia was more frequent in chikungunya-confirmed cases, while myalgia and low back pain were more frequent in dengue-confirmed cases.

We could not accurately describe the proportion of co-infections with arboviruses among the participants. A study carried out in another populous municipality in Brazil by Silva [12] found 9% of co-infections among patients with confirmed dengue, zika or chikungunya.

Malaria corresponded to a large proportion of the total diagnoses performed in the study population, ranked behind only endemic arboviruses. Furthermore, its presence was constant throughout the observation period, that is, it did not vary during outbreaks of other AFIs or seasons of the year. Although, due to the lack of familiarity with the disease, the probability of missing a case of malaria may be higher than a case of leukemia in Brazilian urban health care centers (Lupi, personal communication in Pina-Costa 2014) [13]. Our estimated malaria PPV was twice as high as dengue and zika PPV. As a reference center, malaria microscopy and PCR are part of the standard of care for travelers arriving from an endemic area. Clinical features of malaria and other arboviruses were similar, but chills, sweating and travel history to a transmission area were more common in malaria patients, highlighting the importance of investigating malaria in travelers from endemic areas.

The small number of laboratory-confirmed cases of leptospirosis in this study, may reflect the tendency of thinking about this disease as a cause of AFI only in patients admitted to the hospital with complete Weil´s syndrome, or during rainy seasons [14–16]. Testing routinely for this etiology in outpatients with AFIs may inform the true prevalence of this disease in urban areas of Brazil.

Brazilian spotted fever caused by *Rickettsia rickettsii*, was laboratory confirmed in a limited number of patients as well. The absence of a sensitive test to detect this pathogen during the very first days of symptoms, especially in the mild cases of this disease, might have led to “overtreating” with antibiotics all patients with fever and rash who reported contact with ticks or dense vegetation in areas with large mammals, especially capybaras.

The presence of other arboviruses, such as Mayaro, and Oropuche, often found in the North and Midwest regions of Brazil [17–19] were not investigated in our patients. It is plausible that travelers coming from the Amazon Region, were clinically misdiagnosed as dengue, zika or chikungunya while having Mayaro or Oropuche diseases.

### Patients without diagnosis

During the routine care of patients with AFI, the syndromic classification proved to be an important tool, helping to standardize clinical investigation. However, 41% of the patients in this study were not able to have their clinical presumptive diagnosis confirmed. A large proportion of studies conducted in LMICs described similar proportions of patients without an etiologic diagnosis, even though these studies performed extensive testing for different pathogens [20–24]. In our study, multivariable analysis suggested that respiratory symptoms were the most frequently associated with not having an established etiological diagnosis. It is possible that a large proportion of patients “without diagnosis” had fevers caused by respiratory viral infections which were not tested, and clinical presentation was mistaken for other prevalent diseases.

### Strengths and limitations

A large proportion of travelers from various regions of Brazil and the world were included in our study, with a predominance of those who came from tropical regions. The large number of patients assessed over a long time period permitted that our results were neither deeply affected by seasonality of a given pathogen, nor by outbreaks during the study period. Moreover, a good testing capacity was available. This allowed us to detect the local transmission of emerging infectious diseases such as zika [25,26]. The main limitations of our study were related to incompleteness of clinical records, especially during epidemic periods and the limited availability of POC laboratory diagnosis for opportune investigation.

Finally, syndromic classification is largely used in LMICs to standardize care and guide the patient clinical management when tests availability is limited. In addition to the wider POC diagnostic tests and new technologies implementation, further large-scale studies are needed to develop research algorithms and improve diagnostic accuracy of patients with AFIs.

## Supporting information

S1 Fig Flowchart of specific laboratory tests that may be requested in the first medical visit according to syndromic classification. Abbreviations: EBV = Epstein-Barr Virus, CMV = cytomegalovirus, HIV = human immunodeficiency virus, PCR = polymerase chain reaction, RDT = rapid diagnostic test, CSF = cerebrospinal fluid, CT scan = computed tomography scan, MRI = Magnetic resonance imaging (Adapted from Bressan 2010).
(TIF)

S1 Table Clinical and laboratory criteria used to determine the final diagnosis of study participants according to expert review. (XLSX)
