# Supplementary figures and images for: Challenges of acute febrile illness diagnosis in a national infectious diseases center in Rio de Janeiro: 16-year experience of syndromic surveillance
Source: PLoS Negl Trop Dis. 2023 Apr 3;17(4):e0011232. doi: 10.1371/journal.pntd.0011232 (PMC10101631; doi:10.1371/journal.pntd.0011232)

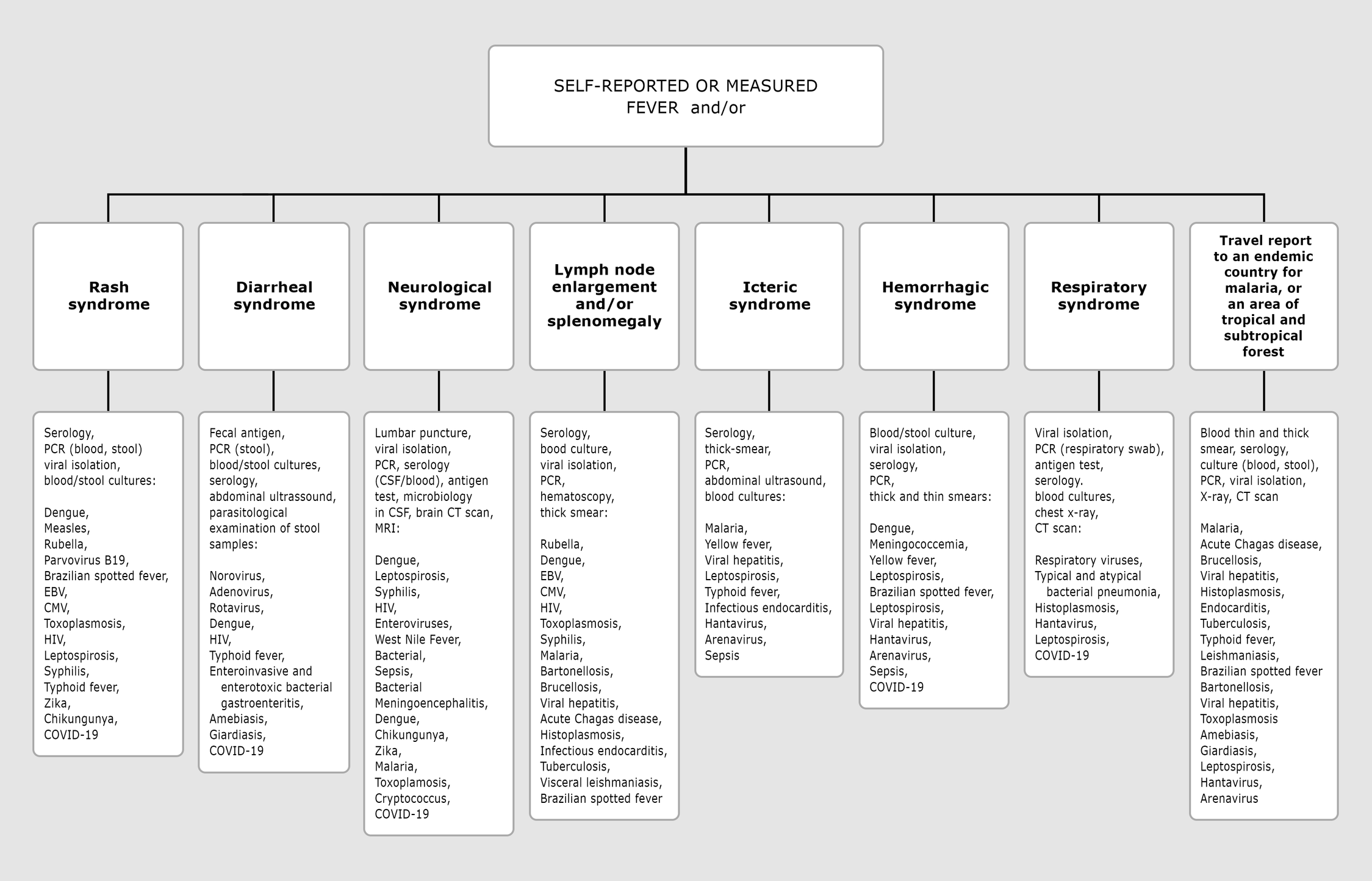

Supplement: S1 Fig — Abbreviations: EBV = Epstein-Barr Virus, CMV = cytomegalovirus, HIV = human immunodeficiency virus, PCR = polymerase chain reaction, RDT = rapid diagnostic test, CSF = cerebrospinal fluid, CT scan = computed tomography scan, MRI = Magnetic resonance imaging (Adapted from Bressan 2010). (TIF) [file pntd.0011232.s001.tif]
